# Dual A_1_ and A_2A_ adenosine receptor antagonists, methoxy substituted 2-benzylidene-1-indanone, suppresses intestinal postprandial glucose and attenuates hyperglycaemia in fructose-streptozotocin diabetic rats

**DOI:** 10.1186/s12902-023-01354-x

**Published:** 2023-05-04

**Authors:** Olakunle Sanni, Gisella Terre’Blanche

**Affiliations:** 1 Centre of Excellence for Pharmaceutical Sciences (Pharmacen), School of Health Sciences, North-West University (NWU), Potchefstroom, 2357 South Africa; 2 Pharmaceutical Chemistry, School of Pharmacy, North-West University (NWU), Private Bag X6001, Potchefstroom, 2520 South Africa

**Keywords:** Adenosine receptor antagonist, hyperglycemia, Glucose tolerance, Glucose absorption

## Abstract

**Background/Aim:**

Recent research suggests that adenosine receptors (ARs) influence many of the metabolic abnormalities associated with diabetes. A non-xanthine benzylidene indanone derivative 2-(3,4-dihydroxybenzylidene)-4-methoxy-2,3-dihydro-1 H-inden-1-one (2-BI), has shown to exhibit higher affinity at A_1_/A_2A_ ARs compared to caffeine. Due to its structural similarity to caffeine, and the established antidiabetic effects of caffeine, the current study was initiated to explore the possible antidiabetic effect of 2-BI.

**Methods:**

The study was designed to assess the antidiabetic effects of several A_1_ and/or A_2A_ AR antagonists, via intestinal glucose absorption and glucose-lowering effects in fructose-streptozotocin (STZ) induced diabetic rats. Six-week-old male Sprague-Dawley rats were induced with diabetes via fructose and streptozotocin. Rats were treated for 4 weeks with AR antagonists, metformin and pioglitazone, respectively. Non-fasting blood glucose (NFBG) was determined weekly and the oral glucose tolerance test (OGTT) was conducted at the end of the intervention period.

**Results:**

Dual A_1_/A_2A_ AR antagonists (caffeine and 2-BI) decreased glucose absorption in the intestinal membrane significantly (p < 0.01), while the selective A_2A_ AR antagonist (Istradefylline), showed the highest significant (p < 0.001) reduction in intestinal glucose absorption. The selective A_1_ antagonist (DPCPX) had the least significant (p < 0.05) reduction in glucose absorption. Similarly, dual A_1_/A_2A_ AR antagonists and selective A_2A_ AR antagonists significantly reduced non-fast blood glucose and improved glucose tolerance in diabetic rats from the first week of the treatment. Conversely, the selective A_1_ AR antagonist did not reduce non-fast blood glucose significantly until the 4th week of treatment. 2-BI, caffeine and istradefylline compared well with standard antidiabetic treatments, metformin and pioglitazone, and in some cases performed even better.

**Conclusion:**

2-BI exhibited good antidiabetic activity by reducing intestinal postprandial glucose absorption and improving glucose tolerance in a diabetic animal model. The dual antagonism of A_1_/A_2A_ ARs presents a positive synergism that could provide a new possibility for the treatment of diabetes.

## Introduction

Postprandial glucose contributed significantly to the management of type 2 diabetes (T2D) [1]. In T2D, the release of insulin is compromised in response to food intake. Insulin resistance leads to the failure of peripheral tissues to utilise postprandial glucose, and results in the development of hyperglycemia [2]. Several reports have established a strong link between hyperglycemia and the development of diabetic complications [2–5]. Hence, the control of postprandial glucose is very crucial to the management of diabetes and its complications. The gastrointestinal absorption of glucose is the major source of postprandial glucose, and the inhibition of this process presents a therapeutic mechanism for diabetes and its complications. Recently, a clinical investigation reported the ability of metformin to reduce the rate of small intestinal glucose absorption in T2D patients, thereby preventing the early onset of diabetic complications [6].

The role of adenosine receptors (ARs) in the regulation of glucose homeostasis and diabetes mellitus has been reported previously, but the specific roles of AR subtypes are still unclear [7–9]. Recently it was indicated that adenosine A_1_, A_2A_, and A_2b_ AR subtypes in rat jejunum contribute to adenosine-mediated vasodilation, with A_2A_ antagonism showing the greatest attenuation [10]. A_2A_ ARs are reported to be the major ARs in intestine epithelial and that its activation increases Cl^−^ secretion [11]. Further, the elevated gene expression level of the A_2B_ ARs has been associated with hyperglycemia in women with gestational diabetes mellitus [12]. Gestational diabetes tends to develop into T2D and cardiovascular disease after pregnancy and is also characterized by chronic insulin resistance. However, there is limited information on the role of A_1_ and A_2A_ ARs in T2D.

Caffeine is a non-selective adenosine antagonist for A_1_/A_2A_ receptors [13]. Epidemiological studies showed that drinking coffee has positive effects on both glucose tolerance and sensitivity to insulin, thus reducing the risk of T2D over long periods of consumption [14, 15]. Another study indicated that caffeine induced a significant reduction in blood sugar (about 65%) in T2D individuals during prolonged low-intensity exercise [16]. In addition, caffeine has shown to stimulate the intestinal anion secretion signalling pathway [17], resulting in membrane depolarisation and reducing the transmembrane Na^+^ force responsible for the Na-dependent uptake of glucose [18]. Further, caffeine was able to reduce small intestinal glucose absorption, increase muscle glucose uptake ex vivo, improve pancreatic β-cell function and stimulate insulin secretions in an animal model of type 2 diabetes [19, 20]. Given the role of endogenous adenosine in various metabolic signalling pathways, the possibility of caffeine and other A_1_ and A_2A_ AR antagonists exerting a contradictory effect on blood glucose levels, could unlock a therapeutic approach toward diabetes.

**Fig. 1:**
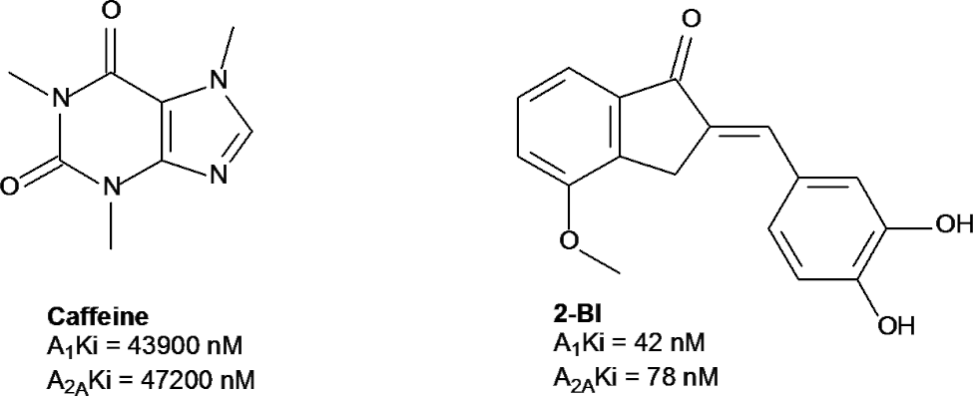
The structure of Caffeine and a non-xanthine benzyl indanone derivative 2-(3,4-dihydroxybenzylidene)-4-methoxy-2,3-dihydro-1 H-inden-1-one (2-BI)

The benzylidene indanone scaffold, containing a C2-phenyl substituted sidechain, has been explored for affinity at A_1_ and/or A_2A_ ARs in rat brains similar to caffeine [21]. A recent study by Janse van Rensburg and co-workers [22] identified a non-xanthine benzylidene indanone derivative 2-(3,4-dihydroxybenzylidene)-4-methoxy-2,3-dihydro-1 H-inden-1-one (2-BI), with dual A_1_/A_2A_ AR affinity in the nanomolar range: A_1_ K_i_ (r) = 42 nM and A_2A_ K_i_ (r) = 78 nM (Fig. 1). The methoxy substituted 2-benzylidene-1-indanone derivatives with C4-OCH_3_ substitution on ring A combined with meta (3′) and para (4′) dihydroxy substitution on ring B on the benzylidene indanone scaffold, showed enhanced A_1_ and A_2A_ affinity [22].

Janse van Rensburg and co-workers (18) predicted the physiochemical and pharmacokinetic properties, as well as the drug-likeness and medicinal chemistry friendliness of 2-BI by using the free web-tool, SwissADME. This compound was considered drug-like as it does not violate any of the rule-based filters of Lipinski, Lombardo, Dominy and Feeney [23], Ghose, Viswanadhan and Wendoloski [24], Veber, Johnson, Cheng, Smith, Ward and Kopple [25] or Egan, Merz and Baldwin [26]. In addition, 2-BI had a bioavailability score of 0.55 (the probability that a compound will have > 10% bioavailability in rats or measurable Caco-2 permeability) [27]. Since 2-BI is a non-xanthine compound, several unwanted effects generally associated with xanthine-containing compounds could be eliminated. These effects include but are not limited to increased gastrointestinal secretions, weak diuretic effects and cardiovascular dysrhythmias [28].

As a result of the higher affinity of 2-BI for both A_1_/A_2A_ AR compared to caffeine, and the structural similarity to caffeine, the current pilot study was initiated to establish the possible antidiabetic effect of 2-BI, by exploring its ability to suppress intestinal glucose absorption and attenuate hyperglycemia in fructose-streptozotocin (STZ) diabetic rats.

## Methods

### In vivo assays

#### Sample preparation

Krebs Buffer Solution comprised of 118 mM NaCl, 5 mM KCl, 1.328 mM, CaCl.2H_2_0, 1.2 mM KH_2_PO_4_, 1.2 mM MgSO_4_, 25 mM NaHCO_3_ at pH 7.4. Sample solutions comprises of different concentrations (0.1 mM, 0.4 mM and 0.8 mM) of caffeine, 2-BI, DPCPX (selective A_1_ AR antagonists), istradefylline (selective A_2_ AR antagonists), and metformin (standard antidiabetic drug) in Kreb buffer containing 11.1 mM of glucose.

#### Animal and tissue preparation

Twelve-week-old male Sprague-Dawley rats were procured from the Vivarium, North-West University, Potchefstroom, South Africa. They were decapitated after fasting for 12 h. The duodenum (~ 10 cm long) were removed and washed in a cold buffer (4.0 °C). All animal protocols were in accordance with the guidelines of the Animal Research Ethics Committee of the North-West University, Potchefstroom, South Africa (ethical approval number: NWU-0041-21-A5).

#### Ussing chamber method

This procedure was done as previously described by Ribi et al. [29] with slight modification. Briefly, the duodenal tissue from each rat was stripped of seromuscular layers, opened and oriented as a flat sheet. The sheet was cut approximately 0.1 cm and vertically mounted on the chamber pin such that the apical membrane was facing one chamber half, whereas the basolateral membrane was facing the other half-chamber, thus separating the solutions that independently bathe each chamber half. Kreb buffer solution (8.0 mL) (pH 7.4) was added to each side of the chamber half. After a pre-incubation period of 20 min, the Kreb buffer in the apical chamber was suctioned by a liquid suction system and immediately replaced by samples of different concentrations. The temperature of the diffuse cells was kept at 37 °C, and the fluids in both compartments were circulated by a stream of O_2_: CO_2_ (95:5.0%). At designated times (0, 15, 30, 45, and 60 min), 200 µL aliquots were taken from the basolateral chamber and replaced with the same volume of Kreb buffer solution. Kreb buffer with 11.1mM was used as a control.

#### Determination of glucose concentration

The glucose concentration of the aliquots was determined using Glucose (GO) Assay Kit from Sigma Aldrich (Catalogue Number GAGO20) following the catalogue procedure.

### In vivo assays

#### Experimental animals

Fifty-six Sprague-Dawley (SD) rats, six-week-old (193 ± 11.94 g), were procured and housed at the North-West University Vivarium, Potchefstroom, South Africa. The male rats were preferred to avoid the physiological variability linked with the oestrous cycle in female rats. The animals were maintained according to the guidelines of the Animal Ethics Regulation Committee (Animcare) of the North-West University with ethics approval number NWU-00529-20-A5).

#### Animal grouping and induction of T2D

Animals were divided randomly into eight groups of seven animals each after one week of acclimatization as shown below.

NC group (citrate buffer, normal control group).

DBC group (fructose + STZ + citrate buffer, diabetic control group).

DBI group (fructose + STZ + 20 mg/kg b.w of 2-BI, dual A_1_ and A_2A_ AR antagonist).

DCA group (fructose + STZ + 20 mg/kg b.w of caffeine, dual A_1_ and A_2A_ AR antagonist) [30].

DIS group (fructose + STZ + 3 mg/kg b.w of istradefylline, selective A_2A_ AR antagonist) [31].

DPX group (fructose + STZ + 0.4 mg/kg b.w of DPXPC, selective A_1_ AR antagonist) [32].

DMF group (fructose + STZ + 300 mg/kg b.w of metformin, standard diabetic drug) [33].

DGT group (fructose + STZ + 10 mg/kg b.w of pioglitazone standard diabetic drug) [34].

Based on its similar structure to caffeine, a dose of 10 mg/kg body weight (b.w.) was used for 2-BI (Kaczmarczyk-Sedlak et al., 2019:1079–1080). Results from a MTT toxicity assay on the viability of cultured HepG2 cells. showed low cytotoxicity of 2-BI with an IC_50_ value of 399.6 µM (reference to Jankowitz, 2023). Both metformin and pioglitazone were included since these drugs are used for the treatment of T2D, and because their mode of action differs. Metformin belongs to a group of medicines called biguanides and exerts its effect primarily by decreasing hepatic glucose output. Pioglitazone belongs to a group of medicines called thiazolidinediones and improves glycemic control primarily by increasing peripheral insulin sensitivity [35].

#### Induction of diabetes

Animals in diabetic groups were induced as previously described by Sanni et al., [36]. Briefly, animals in diabetic groups (DBC, DBI, DCA, DIS, DPX DMF and DGT) were given 10% fructose solution ad libitum for two weeks to induce insulin resistance followed by a single intraperitoneal injection of streptozotocin (STZ) (40 mg/kg b.w dissolved in citrate buffer pH 4.5) to cause partial destruction of pancreatic β-cells. Animals in the normal control group (NC) were given water ad libitum for two weeks followed by a single intraperitoneal injection with citrate buffer (40 mg/kg b.w) instead of 10% fructose and STZ respectively. After a week, the non-fasting blood glucose (NFBG) of all the animals was measured from the tail vein using a portable glucometer (Glucoplus Inc. Canada). Animals with NFBG greater than 11 mmol/L were considered diabetic, while the animals with NFBG less than 11 mmol/L, were excluded from the diabetic groups. However, not all the animals were diabetic, and the groups were randomly rearranged containing six animals instead of seven animals per group, with a mean of 15.02 ± 1.04 blood glucose in each diabetic group.

#### Intervention period

After the confirmation of diabetes (2.2.3 above), the animals were given their respective dose (as shown in the animal grouping) everyday for 4 weeks using a gastric gavage needle. The respective doses were determined from previous research using animal model of rats as indicated in the animal grouping. However, the dose of 2-BI was determined based on the dose of caffeine because of their structural and AR receptor similarity. Animals in the control group were treated with the same volume of the vehicle. During the intervention period, daily fluid and food intake were measured and body weight and NFGB were measured weekly in all the groups.

#### Oral glucose tolerance test (OGTT)

OGTT was performed in the last week of the intervention period. The animals were given an oral dose of glucose solution (2 g/kg b.w) after an overnight (12 h) fasting. The blood glucose levels were measured at 0 (just before glucose ingestion), 30, 90, and 120 min after the glucose ingestion using a portable glucometer (Glucoplus Inc., Quebec, Canada). The area under the curve (AUC) was calculated according to the method as described by Turner [37].

#### Serum insulin concentration

The serum insulin concentration was determined using Rat Ins1 / Insulin ELISA Kit from Sigma Aldrich (Catalogue Number RAB0904-1KT) following the catalogue procedure.

#### Homeostatic model assessment

Homeostatic model assessment scores are used to determine of the pancreatic β-cell function (HOMA- β) and insulin resistance (HOMA- IR) from fasting serum insulin and glucose concentrations. These were calculated according to the following formula:

HOMA-IR = [Fasting serum insulin (U/L) * Fasting blood glucose (mmol/L)] /22.5]

HOMA-β = [20 * Fasting serum insulin (U/L)]/ Fasting blood glucose (mmol/L) -3.5 Conversion factor: insulin (1U/L = 7.174 pmol/L).

#### Liver glycogen estimation

Liver glycogen was estimated according to the method of Lo et al. [38] with slight modification. Briefly, 0.3 g of the liver tissue was cut and digested with 0.5 mL of 30% potassium hydroxide saturated with sodium sulphate (Na_2_SO_4_). The resulting solution was boiled for 30 min and immediately cooled on ice. Thereafter, 670 µL of 95% ethanol was added and allowed to stand in ice for 30 min and centrifuged at 840 g for 30 min to precipitate the glycogen. The supernatant was discarded, and the residue was then re-suspended in 300 µL 0f 95% ethanol and centrifuge for 20 min to further precipitate the glycogen. The glycogen precipitate obtained was dissolved in 1 mL of distilled water. An aliquot of 20 µL was taken and made up to 200 µL with distilled water in a tube. Thereafter, 200 µL of 5% phenol was added to the aliquot and the glycogen standards (10, 20, 30, 40, 50, 60, 70, 80, 90 µg/mL) followed by 1 ml of sulphuric acid (96–98%). They were boiled in a water bath for 10 min. The tubes were allowed to cool for 10 min and absorbance read at 490 nm in a plate reader (Synergy HTX Multi-mode reader, Bio Tek Instrument Inc. Winooski, USA). Glycogen content was calculated from the glycogen standard curve using a log-log graph and expressed as µg/mg.

### Statistical analysis

Data were represented as means ± SD and analysed with a one-way ANOVA using Windows SPSS statistical software package (version 26, IBM Corporation, New York, USA) and Prism 8 (Version 1.0), followed by Tukey’s-HSD multiple range post hoc tests. P < 0.05 was considered statistically significant.

## Results

### Effect of A_1_ and A_2A_adenosine receptor antagonists on intestinal glucose absorption

**Fig. 2:**
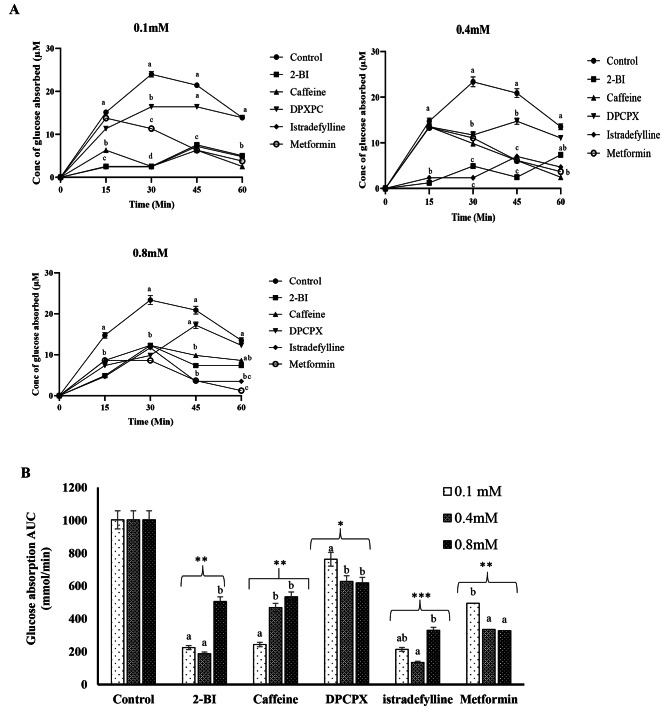
**(A)** Effect of A_1_ and A_2_ AR antagonist on glucose absorption across rat jejunum segment isolated in Ussing chamber. AR antagonists were prepared in Kreb buffer containing 11.1mM of glucose was added on the mucosal side. **(B)** AUC data for 0–60 min post glucose absorption are shown. Baseline subtraction for control was used to calculate AUC values for all groups, to ensure full treatment benefits are recognised. Values represent mean ± SD for triplicate. ^a−c^ Different alphabets near the lines (2 A) or bar (2B) for a given time or group respectively represent the significance of difference (p < 0.05). *P < 0.05, **P < 0.01 and ***P < 0.001 compared to control

Glucose absorptions were observed for one hour and progress was monitored at 15, 30, 45 and 60 min. The control showed a steady increase in the amount of glucose absorbed and peaked at 30 min and showed a decline at 45 and 60 min (Fig. 2A), thereby indicating that absorption occurred from the apical to the basolateral membrane. These results are in accordance with the mechanism of glucose absorption across the intestinal through SGLT1 located at the apical surface of enterocytes [18]. A_1_/A_2A_ AR antagonist as well as the standard drug, metformin, also showed a similar pattern compared to the control either peaking at 15 or 30 min, however, the concentration of glucose absorbed in these groups was lower than the control.

Using area under the curve (AUC) (Fig. 2B), the effect of A_1_/A_2A_ AR antagonists on intestinal glucose absorption was compared to the control. All the treatments, A_1_/A_2A_ AR antagonists and metformin, decreased glucose absorption significantly in the rat jejunum compared to the control. Caffeine and 2-BI, both dual A_1_/A_2A_ AR antagonists, decreased the glucose absorption by the apical membrane significantly (p < 0.01) and exerted similar decreasing effects at a concentration of 0.1mM, while 2-BI showed a higher decrease in glucose absorption at 0.4 mM. DPXPC, an A_1_ AR selective antagonist, showed the lowest significant (p < 0.05) decrease in glucose absorption at all concentrations compared to the control, and thus not as effective as the dual A_1_/A_2a_ AR antagonism of caffeine and 2-BI. Istradefylline, a selective A_2A_ AR antagonist, showed the highest significant (p < 0.001) decrease in glucose absorption compared to the control. Istradefylline showed a similar decrease in glucose absorption compared to 2-BI at 0.1 and 0.4 mM concentrations and a much higher decrease in glucose absorption at a concentration of 0.8 mM. Istradefylline showed a much higher decrease in glucose absorption compared to DPCPX and thus implying that A_2A_ AR antagonism may play a larger role in decreasing glucose uptake by the apical membrane than A_1_ AR antagonism. Metformin significantly (p < 0.01) decreased glucose absorption in a dose-dependent manner compared to the control, and its effect compared well to that of istradefylline and 2-BI. Taking the effect of concentration into consideration, it was obvious that only DPXPC and metformin demonstrated a dose-dependent decrease in glucose absorption (Fig. 2B).

### In vivo effect of A_1_ and A_2a_ adenosine receptor antagonists on non-fasting blood glucose (NFBG)

**Fig. 3:**
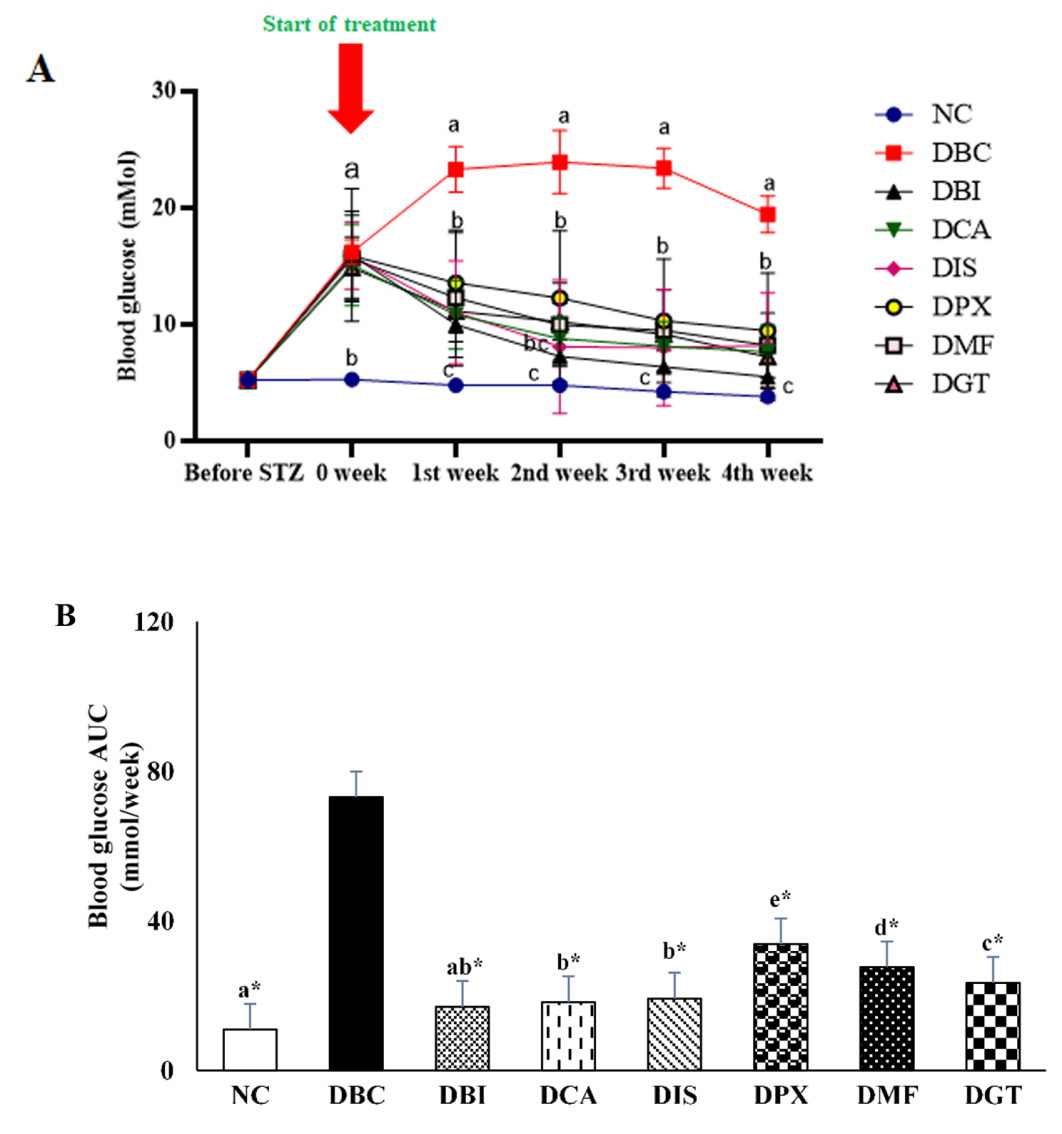
**(A)** glucose-lowering effect of A_1_ and A_2_ AR antagonist on non-fasting plasma glucose measured before diabetes induction and throughout the weeks of intervention. **(B)** AUC data for 0–4th week blood glucose are shown. Baseline subtraction for normal control was used to calculate AUC values for all groups, to ensure full treatment benefits are recognised. Values represent mean ± SD for 6 rats. ^a−e^ Different alphabets near the lines for a given time (3 A) or bar (3B) for a given time or group respectively represent significance of difference (p < 0.05). * significance of difference P < 0.05 compared to Diabetic control group. NC, Normal Control; DBC, Diabetic Control; DBI, Diabetic + 2-BI; DCA, Diabetic + caffeine; DIS, Diabetic + istradefylline; DPX, Diabetic + DPXPC; DBM, Diabetic Metformin; DGT, Diabetic + pioglitazone

NFBG was monitored weekly in fructose-STZ diabetic rats. After five days of diabetic induction, the NFBG increased significantly (P < 0.05) in all the diabetes groups (DBC, DBI. DCA, DIS, DPX, DMF, and DGT) compared to the normal control group (NC)(Fig. 3A). This increase in blood glucose showed that diabetes was successfully induced in the diabetic groups. The diabetic rats treated with dual A_1_/A_2A_ AR antagonists (DBI and DCA) and a selective A_2A_ AR antagonist (DIS) significantly reduced NFBG from the first week of the intervention when compared to the DBC. However, diabetic rats treated with a selective A_1_ AR antagonist (DPX) did not reduce NFBG significantly until the 4th week of the intervention when compared to the DBC, thus once again confirming A_2A_ AR antagonism plays a larger role than A_1_ AR antagonism in glucose control. Interestingly, all the treatment groups showed similar blood glucose levels compared to the NC group at the end of the intervention period. Comparing the AUC data for 0–4 weeks of blood glucose levels (Fig. 3B), it was also observed that the treatment groups DBI, DCA, and DIS, had a larger effect in reducing NFBG than the DPX,

DMF, and DGT groups compared to the diabetic control group (DBC), which may imply a better reduction of non-fasting blood glucose with a dual A_1_/A_2A_ AR antagonist.

### In vivo effect of A_1_ and A_2a_ adenosine receptor antagonists on oral glucose tolerance test (OGTT)

**Fig. 4:**
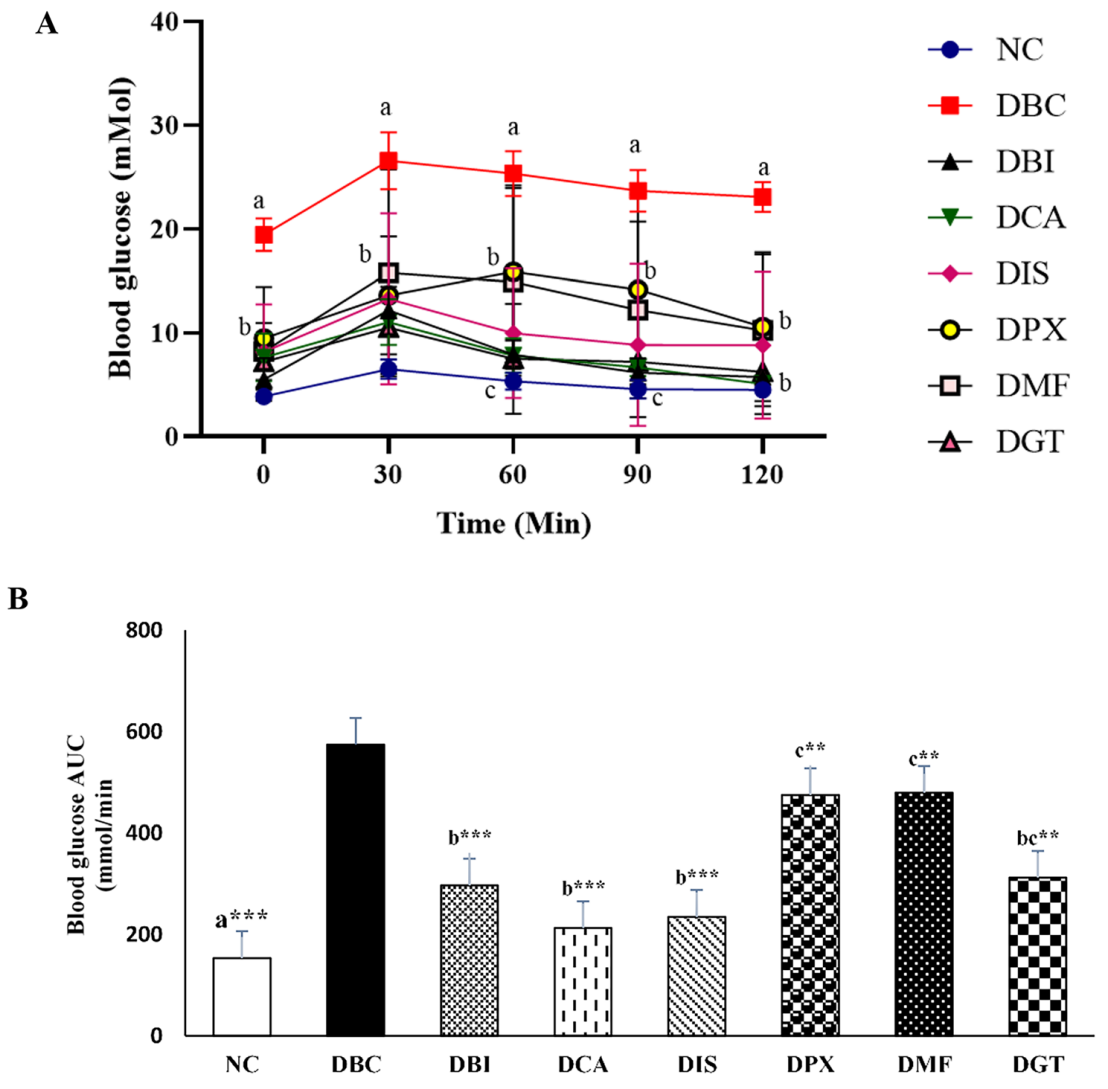
**(A)** Effects of A_1_ and A_2_ AR antagonist on glucose tolerance. After the 24-day treatment period, Rats were fasted (18 h) overnight and blood glucose was measured at 0. 30, 90, and 120 min after the oral administration of glucose solution (2 g/kg b.w). **(B)** AUC data for 0–120 min post glucose injection were shown. Baseline subtraction for control mice was used to calculate AUC values for all groups, to ensure full treatment benefits are recognised. Values represent mean ± SD for 6 rats. ^a−c^ Different alphabets near the lines (4 A) or bar (4B) for a given time or group respectively for a given time represent significance of difference (p < 0.05). **P < 0.01 and ***P < 0.001 compared to Diabetic control group. NC, Normal Control; DBC, Diabetic Control; DBI, Diabetic + 2-BI; DCA, Diabetic + caffeine; DIS, Diabetic + istradefylline; DPX, Diabetic + DPXPC; DBM, Diabetic Metformin; DGT, Diabetic + pioglitazone

Following the 4-week intervention period, an oral glucose administration and tolerance test for 120 min were done with 30 min intervals (Fig. 4A). All treatment groups showed an improvement in glucose tolerance when compared to the diabetic control group (DBC). Comparing the AUC data (Fig. 4B) for 0–20 min post glucose injection, glucose levels were significantly (P < 0.05) decreased in all the treatment groups when compared to DBC. The test compound (DBI), caffeine (DCA), and istradefylline (DIS) were more effective in improving glucose tolerance (p < 0.001) when compared to DPCPX (p < 0.05 DPX), and the standard drug treatment groups metformin (p < 0.05) and pioglitazone (p < 0.01 DGT). Further DBI, DCA and DIS followed the same trend and the normal control (NC). As expected, the DMF group demonstrated a lesser effect when compared to all the treated groups and significantly (P < 0.05) poor in improving glucose tolerance when compared to the normal control group (p < 0.001).

### Effect of A_1_ and A_2A_adenosine receptor antagonists on serum insulin concentration

The serum insulin concentration was determined at the end of the study. The low serum insulin concentration in the diabetic control group indicated pancreatic dysfunction, therefore signifying diabetic condition. However, treatment with the different antagonists of A_1_ or A_2A_ AR and standard drugs (metformin and pioglitazone) significantly improved the serum insulin concentration. Within the treatment groups, the dual A_1_/A_2A_ AR antagonists (DBI and DCA) demonstrated the significant (P < 0.05) highest insulin concentration in the serum compared with both specific A_1_ (DPX) and A_2A_ (DIS) AR antagonists (Table 1).

### Effect of A_1_ and A_2A_adenosine receptor antagonists on liver glycogen content

The liver glycogen content was significantly (P < 0.05) depleted in the diabetic control group, but the treatment with the different antagonists of A_1_ or A_2A_ AR and standard drugs (metformin and pioglitazone) significantly (P < 0.05) improve the glycogen content (Table 1). The dual A_1_/A_2A_ AR antagonists (DBI and DCA) demonstrated the highest glycogen content as compared to specific A_1_ (DPX) and A_2A_ (DIS) AR thus suggesting the involvement of both A_1_ and A_2A_ AR in the glycogenesis in response to insulin. Interestingly, DIS and DPX demonstrated a similar response to the liver glycogen content.

**Table 1:** In vivo effect of A_1_ and A_2a_ Adenosine Receptor Antagonists on serum insulin concentration, liver glycogen content and Homeostatic assessment model

	Serum insulin concentration (ρmol/L)	Homeostatic assessment model		Liver glycogen content (mg/g tissue)
		HOMA-IR	HOMA-β	
NC	70.63 ± 1.45^a^	1.65 ± 0.09^a^	313 ± 10.43^a^	20.87 ± 1.92^a^
DBC	34.51 ± 2.34^e^	3.86 ± 0.45^e^	5.6 ± 0,34^f^	4.24 ± 1.10^e^
DBI	58.17 ± 3.26^b^	1.84 ± 0,1^b^	75.76 ± 11.67^b^	17.83 ± 1,14^b^
DCA	45.90 ± 2,18^d^	2.02 ± 0.87^bc^	28.73 ± 6.23^d^	14.63 ± 1.39^c^
DIS	49.83 ± 1.04^ cd^	2.19 ± 0.76^c^	31.21 ± 5.75^d^	9.68 ± 0.58^d^
DPX	40.80 ± 2.02^e^	2.73 ± 0.35^ cd^	13.05 ± 0.95^e^	9.44 ± 0.74^d^
DMF	44.96 ± 3.45^de^	2.4 ± 0.98^d^	19.3 ± 9.45^e^	13.11 ± 1.03^c^
DGT	52.75 ± 2.97^c^	1.88 ± 0.23^b^	50.47 ± 2.74^c^	13.55 ± 1.46^c^

Results are expressed as mean ± SD of six rats. ^a−e^ Different superscripts alphabets down the column indicate the significant difference (Tukey’s-HSD multiple range post hoc test, P < 0.05)

NC, Normal Control; DBC, Diabetic Control; DBI, Diabetic + 2-BI; DCA, Diabetic + caffeine; DIS, Diabetic + istradefylline; DPX, Diabetic + DPXPC; DBM, Diabetic Metformin; DGT, Diabetic + pioglitazone

### Effect of A_1_ and A_2A_adenosine receptor antagonists on the homeostatic assessment model

The homeostatic assessment model, both HOMA-IR and HOMA-β, were significantly (p < 0.05) higher in diabetic groups than in the control group. Furthermore, the HOMA-IR index was significantly (p < 0.05) lower in control the dual A_1_/A_2A_ AR antagonists (DBI and DCA) as compared with the specific A_1_ (DPX) and A_2A_ (DIS) AR. When compared with the standard drug, pioglitazone, the dual A_1_/A_2A_ AR antagonists show no significant differences (p < 0.05). This observation indicates the improved insulin resistance in the diabetic groups with the treatment of the A_1_ and A_2A_ARAntagonists. The HOMA-β is an index for β-cell functions and was significantly (p < 0.05) low in the DBC. Treatment with A_1_ and A_2A_ARAntagonists significantly improved the β-cell functions as indicated in their HOMA-β (Table 1). The dual A_1_/A_2A_ AR antagonist, DBI, significantly improved the β-cell functions as compared with other treatments. This observation further supports the synergist effect of A_1_and A_2A_ AR antagonists in glucose homeostasis.

## Discussion

In the present study, we demonstrated the role of adenosine receptor antagonists in the intestinal absorption of glucose in the jejunal of normal, Sprague-Dawley rats. The intestinal absorption of glucose contributes significantly to postprandial hyperglycemia and therefore, inhibition of this process presents a therapeutical approach. The mechanism of intestinal glucose absorption involves the sodium-dependent hexose interactions with jejunal and ileal enterocyte glucose transporters (SGLT1) in the apical which actively transports glucose from the lumen of the intestine into enterocytes [39]. Once transported into the cell, glucose is carried across the basolateral membrane of the epithelial cell through the GLUT2 transporter [40] which provides an exit pathway of glucose from the cytosol into the blood.

Adenosine causes a rapid increase in carrier-mediated glucose uptake that is of clinical relevance and acts via receptors linked to a signalling pathway that involves intracellular cAMP production [41]. Previous work has indicated that increased intracellular cAMP can increase both the enterocyte membrane potential, and thus the driving force for Na+-glucose co-transport, and the rate of glucose uptake across both the brush border and basolateral membranes [42]. Recently, the increased levels of cAMP were found to increase the cell surface expressions of Na+/K+-ATPase and cAMP-dependent vesicle trafficking like protein kinase A (PKA) which may regulate SGLT1 exocytosis and insertion into the apical membrane [43]. It has been reported that A_1_ and A_3_ ARs inhibit cAMP production, while A_2A_ and A_2B_ ARs stimulate cAMP [42].

In the current study, selective A_2A_AR antagonist, istradefylline and dual A_1_/A_2A_ AR antagonists, caffeine and 2-BI, significantly reduced the intestinal glucose absorption, while selective A_1_AR antagonist, DPCPX, was not as effective and thus suggests a larger involvement of A_2A_ AR antagonism than A_1_ AR antagonism. Our observation is similar to that of Li and co-workers who suggested the adenosine A_2A_ ARS to be partially involved in glucose-induced vasodilation of the jejunum, responsible for a local microvascular mediator that could be associated with the control of blood flow during intestinal absorption of nutrients [10].

The role of A_1_ and A_2A_ AR antagonists in the maintenance of blood glucose has not been thoroughly investigated. Caffeine, a dual A_1_/A_2A_ AR antagonist, has been widely investigated for its anti-hyperglycemia effects in diabetic animals with contradictory reports. This suggests the possibility of different mechanisms that might be involved in the antagonism of A_1_ and A_2A_ ARs. Therefore, the antidiabetic activity of the novel dual A_1_/A_2A_ AR antagonist, 2-BI, was explored and compared to the known dual A_1_/A_2A_ AR antagonist, caffeine, selective A_2A_ AR antagonist, istradefylline and selective A_1_ AR antagonist, DPCPX, in fructose-STZ diabetic rats.

Hyperglycemia is the hallmark of T2D and maintaining glucose hemostatic has been the major target for the prevention of hyperglycemia and its associated complications. There are different therapeutical approaches in lowering high blood glucose in the body, which include decreasing hepatic glucose production, increasing the sensitivity of peripheral tissue towards the action of insulin, and increasing the functionality of pancreatic β-cells. As expected, both metformin and pioglitazone reduced the high blood glucose in diabetic rats (Fig. 3). Metformin works majorly by inhibiting hepatic gluconeogenesis and opposing the action of glucagon, while pioglitazone works by increasing the sensitivity if the cell towards insulin action [44]. Adenosine has also been shown to increase gluconeogenesis and glycogenolysis via increasing cAMP by stimulation of hepatic adenylate cyclase through e A_2A_ AR binding in the liver. Both or either of these actions causes an increase in local insulin resistance and glucose output from the liver [45]. As a result, the antagonism of A_2A_ ARs is expected to oppose these actions, and hence, reduce insulin resistance and glucose output from the liver. A recent study also indicated that caffeine stimulates A_2A_ ARs and triggers glycogenesis in the liver [46]. Interestingly, the dual A_1_/A_2A_ AR antagonists, 2-BI and caffeine, and the selective A_2A_ AR antagonist, istradefylline, demonstrated an improved anti-hyperglycemic activity compared to metformin and pioglitazone.

A_1_ AR selective agonist, 2-chloro N^6^ -cyclopentyladenosine (CCPA), has been shown to alleviate diabetes by increasing insulin = stimulated glucose transport in isolated rat muscle [47, 48], and thus ruled out the possible protective effect of A_1_ AR antagonist against diabetes. However, the current findings are contradictory, for DPCPX (selective A_1_ AR antagonist) reduced high blood glucose in diabetic rats and could be the result of other mechanisms apart from glucose transport in the muscle. A similar study indicated that A_1_ AR activation inhibits insulin release from the rat pancreatic β-cell line INS-1 and rat islets [47]. Therefore, the antagonist effect of A_1_ AR could possibly activate the insulin release from the pancreatic β-cell. This assumption was further supported by Xu and co-workers who observed that oral administration of selective A_1_ AR antagonist, BW-1433, increased insulin sensitivity in Zuker rats [49].

The liver glycogen depot plays an important role in keeping up blood glucose homeostatic processes in the postprandial state which is regulated by insulin action and sensitivity. Disruption in hepatic glycogen metabolism as a result of metabolic dysfunction by insulin insensitivity has been associated with type 2 diabetes mellitus (T2D). A Recent study has demonstrated the contributing role of adenosine receptors to glycogenesis in rat hepatocytes. A_2A_ AR antagonist, 8-(3-chlorostyryl)caffeine (CSC), and A_1_ AR selective antagonist, (+)®[(E)-3-(2-phenylpyrazolo[1,5-alpha]pyridin-3-yl)acryloyl]-2-piperidine ethanol (FK453), have been shown to inhibit glycolysis and gluconeogenesis but improve glycogenesis in the hepatocytes [50]. In the present study, we observed the same trend in the glycogen content in the dual A_1_/A_2A_ antagonist, 2-BI and caffeine, which improved the glycogen content in the liver of diabetic rats.

The HOMA model is used to determine an estimate of insulin sensitivity and β-cell function from fasting blood glucose and plasma insulin concentrations. The connection between glucose and insulin in the basal state mirrors the equilibrium that exists between hepatic glucose output and insulin secretion, which is regulated by a feedback mechanism between the liver and β-cell [51]. The HOMA model assessment in our present study further support that the liver glycogen content is a function of both insulin secretion (as in HOMA- β) and insulin resistance (as in HOMA-IR). The 2-BI improved both the insulin secretion and same time increased the cell sensitivity to the action of insulin in diabetic rats. However, Masaru and his co-worker observed that the activation of A_2A_ AR by A_2A_ agonist (ATL-146e) attenuates inflammation in animal models [52]. Considering the role of inflammation in the aetiology of insulin resistance, one may assume that A_2A_ antagonists play little or no significant role in improving insulin resistance in the liver. Furthermore, a recent study on the role of A_2A_ AR in the whole-body insulin sensitivity in a prediabetes animal model, concluded that A_2A_ adenosine antagonists restored impaired insulin signaling in the skeletal muscle of HSu rats, but did not affect the liver or adipose insulin signaling [53]. Our result strengthens this hypothesis as can be seen in the HOMA-IR results of the selective A_2A_ AR antagonist, istradefylline (DIS group, Table 1). The improved insulin sensitivity observed in our study in the DIB group could be attributed to the insulin sensitivity in the skeletal muscle rather than the liver considering the non-significant difference in the liver glycogen content among the AR antagonist treated groups (DIB, DCA, DIS, and DPX).

Contradictory, Töpfer and co-workers [54] observed that caffeine (nonselective AR A_1_ /A_2A_ antagonist) increased insulin release and suggested that both receptors could be involved and not necessarily be coupled to the opening of potassium channels (^86^Rb^+^ efflux experiments) or inhibition of calcium channels. Our findings in this study complement this observation as we observed increased insulin serum concentration in both the DCA group (caffeine) and the DIB group (2-BI).

Overall, the anti-hyperglycemia potential demonstrated by 2-BI in our study could be a result of simultaneous action or synergism of A_1_ and A_2A_ AR antagonism in key organs such as the liver, adipose tissue, muscle and pancreas in glucose and lipid homeostasis. For example, the increase in serum insulin, increase in liver glycogen content, and consequence improvement in the homeostasis assessment model as witnessed in the DBI and DCA group correlate with the reduction in high blood glucose in the diabetic group treated with DBI and DCA. Also, the involvement of A_1_ /A_2A_ AR antagonist in insulin release and the improvement of insulin sensitivity (as shown in the HOMA- β and HOMA- IR respectively) could be the underlying mechanism of its antihyperglycemic potential. However further studies are required to unravel these mechanisms.

## Conclusion

The role of adenosine receptors, especially A_1_ and A_2A_, in glucose and lipid homeostasis present an emerging field for a new therapeutic approach toward diabetes and its associated complication. The novel compound, 2-BI, demonstrated the potential to be considered as a candidate for a new strategy for diabetes treatment. The dual A_1_/A_2A_ AR antagonist, 2-BI, exhibited good antidiabetic activity in vitro by reducing intestinal postprandial glucose absorption and in vivo by improving glucose tolerance in a diabetic animal model. 2-BI had similar antidiabetic effects compared to caffeine and istradefylline and obtained even better results than current T2D treatments, metformin and pioglitazone. Beyond doubt, our results support the growing evidence that A_1_ and A_2A_ ARs play a significant role in glucose homeostasis. However, the positive synergism of dual A_1_/A_2A_ AR antagonism on glucose absorption and tolerance do require further investigation.

## Data Availability

The datasets used and/or analysed during the current study available from the corresponding author on reasonable request.
